# Transmucosal Solid Lipid Nanoparticles to Improve Genistein Absorption via Intestinal Lymphatic Transport

**DOI:** 10.3390/pharmaceutics13020267

**Published:** 2021-02-16

**Authors:** Antonella Obinu, Giovanni Pietro Burrai, Roberta Cavalli, Grazia Galleri, Rossana Migheli, Elisabetta Antuofermo, Giovanna Rassu, Elisabetta Gavini, Paolo Giunchedi

**Affiliations:** 1 Department of Chemistry and Pharmacy, University of Sassari, via Muroni 23/a, 07100 Sassari, Italy; aobinu@uniss.it (A.O.); grassu@uniss.it (G.R.); pgiunc@uniss.it (P.G.); 2 Department of Veterinary Medicine, University of Sassari, 07100 Sassari, Italy; gburrai@uniss.it (G.P.B.); eantuofermo@uniss.it (E.A.); 3 Mediterranean Center for Disease Control (MCDC), University of Sassari, 07100 Sassari, Italy; 4 Department of Science and Technology of Pharmaceutics, University of Torino, 10125 Torino, Italy; roberta.cavalli@unito.it; 5 Department of Medical, Surgical and Experimental Sciences, University of Sassari, 07100 Sassari, Italy; galleri@uniss.it (G.G.); rmigheli@uniss.it (R.M.)

**Keywords:** solid lipid nanoparticles, genistein, oral bioavailability, intestinal lymphatic absorption

## Abstract

Genistein (GEN) is a soy-derived isoflavone that exhibits several biological effects, such as neuroprotective activity and the prevention of several types of cancer and cardiovascular disease. However, due to its poor water solubility and the extensive first-pass metabolism, the oral bioavailability of GEN is limited. In this work, solid lipid nanoparticles (SLN) were developed to preferentially reach the intestinal lymphatic vessels, avoiding the first-pass metabolism of GEN. GEN-loaded SLN were obtained by a hot homogenization process, and the formulation parameters were chosen based on already formulated studies. The nanoparticles were characterized, and the preliminary in vitro chylomicron formation was evaluated. The cell uptake of selected nanocarriers was studied on the Caco-2 cell line and intestinal mucosa. The SLN, characterized by a spherical shape, showed an average diameter (about 280 nm) suitable for an intestinal lymphatic uptake, good stability during the testing time, and high drug loading capacity. Furthermore, the intestinal mucosa and Caco-2 cells were found to uptake SLN. The approximately two-fold increase in particle size suggested a possible interaction between SLN and the lipid components of chylomicrons like phospholipid; therefore, the results may support the potential for these SLN to improve oral GEN bioavailability via intestinal lymphatic absorption.

## 1. Introduction

The lymphatic system is part of the circulatory system, playing a significant role in the immune system and in the maintenance of the liquid balance in the body: it collects excess fluid and plasma proteins from the interstitial fluid and deposits them in the bloodstream. However, lymphatic vessels represent a primary method of dissemination for infectious agents and metastatic tumor cells all throughout the body [1,2]. Therefore, the lymphatic route has been used for the delivery of drugs to treat diseases that spread via the lymphatic system, like cancer and the human immunodeficiency virus [3]. The gastrointestinal tract is rich in lymphatic and blood vessels, representing an interesting opportunity for the site-specific absorption of lipid drugs, proteins, vaccines, and peptides following oral administration [4].

Solid lipid nanoparticles (SLN) are suitable carriers to deliver drugs to specific intestinal associated structures, such as lymph nodes and lymphatic vessels [5]. Indeed, they show features that make them suitable candidates for intestinal lymphatic drug transport via the oral route [6]. The small size of these nanocarriers ensures an efficient lymphatic intestinal absorption that is reported to be exclusive for particles ranging from 20 to 500 nm in size [3]. Furthermore, it has been well demonstrated that oral lipid nanocarriers can allow for the formation of chylomicrons by enterocytes that facilitate the uptake of the lipid matrix by intestinal lymphatic vessels [7,8].

Genistein (GEN) is an isoflavone characterized by several pharmacological properties. In the last decades, GEN has drawn a great deal of attention for its use in the treatment and prevention of many diseases, including several types of tumors [9,10,11], cardiovascular diseases [12], osteoporosis, and hormonal pathologies [13]. Besides this, several studies have confirmed the neuroprotective effect of this drug [14,15,16]. However, despite encouraging in vitro and in vivo results, the clinical use of GEN is limited due to its disadvantages. GEN is sensitive to oxidation, heat, and light, and suffers from extensive first-pass metabolism, poor oral bioavailability due to low water solubility (<1µg/mL), and reduced permeability through the apical surface of intestinal cells [17,18,19]. Therefore, to improve the GEN pharmacokinetic profile, suitable drug delivery systems are needed [20,21,22].

The overall aim of this study was to develop transmucosal SLN characterized by a high loading capacity and the ability to preferentially reach the intestinal lymphatic vessels. Due to the direct drainage of intestinal lymphatic vessels into the circulatory system, the first-pass metabolism of GEN can be avoided [23]. At the same time, through the lymphatic vessels, GEN can reach the lymph node metastases, where it could contribute to the suppression of cancer cells.

Several preliminary studies were carried out to identify a suitable formulation for GEN entrapment and, after preparation, the SLN were characterized in vitro and ex vivo.

A solid lipid matrix was selected for SLN preparation: Compritol 888 ATO (glyceryl behenate) (C), a lipophilic excipient generally recognized as safe (GRAS) by FDA [24]. In this study, to investigate SLN cellular uptake, the human intestinal cell culture Caco-2 was selected. As previously described, the Caco-2 cell line provides an interesting intestinal barrier model for the assessment of bioaccessibility, intestinal uptake, and bioavailability in bioactive compounds [25,26].

## 2. Materials and Methods

### 2.1. Materials

Synthetic GEN (98% pure as determined by HPLC) was purchased from Farmalabor (Milan, Italy); C was obtained from Gattefossè (Saint-Priest, France). Tween 80 was purchased from Acros Organics (Geel, Belgium). Acetonitrile and methanol were of chromatographic grade and were purchased from Merck (Darmstadt, Germany). Phosphatidylcholine derived from soybeans (Lipoid S100) was a kind gift from Lipoid GmbH (Ludwigshafen, Germany). Acetic acid and pancreatin from the porcine pancreas were purchased from Sigma-Aldrich (Steinheim, Germany). Fluorescein was purchased from Fluka Analytical (Buchs, Switzerland). Acetone, KH_2_PO_4_, and NaOH were obtained by Carlo Erba (Milan, Italy). Ultrapure bidistilled water was obtained by a MilliQ R4 system, Millipore (Milan, Italy). The fresh intestine was taken from a local slaughterhouse (Forma s.r.l; CE IT D2L5B (Regulation EC 853/2004)). Paraffin, hematoxylin, and eosin were purchased from Bio-Optica (Milan, Italy). Dulbecco’s modified Eagle’s medium (DMEM/F12, HEPES, no phenol red) was acquired from Life Technologies (Monza, Italy). Phosphate-buffered saline ((PBS), 0.2 µm filtered, adjusted to pH 7.4) solution and Trypan Blue (0.4%) were purchased from Sigma-Aldrich.

### 2.2. Preparation of Unloaded SLN

Based on extant studies reported in the supplementary material, a blank SLN formulation (B-SLNc) was prepared. C (2.0% *w*/*v*) was weighed and melted at 80 °C. Tween 80 was dissolved in distilled water to have a final concentration of 0.5% (*w*/*v*) and heated at 85 °C. The homogenous lipid phase was added to the hot aqueous surfactant solution under magnetic stirring to obtain an oil/water (O/W) pre-emulsion. The final emulsion was obtained via homogenization using the probe sonicator at 50% amplitude for 12 min, maintaining the temperature at 85 °C to avoid solidification of the lipid. The nanoemulsion obtained was then cooled in an ice bath for 20 min under constant magnetic agitation to achieve SLN dispersion in water.

### 2.3. Preparation of GEN-Loaded SLN

Genistein-loaded SLN were prepared (Table 1). Briefly, GEN (0.02, 0.03, 0.04, and 0.06 *w*/*v*) was mixed with C (2.0% *w*/*v*), and the molten lipid phase (80 °C) was added to a 0.5% (*w*/*v*) Tween 80 aqueous solution that had been previously heated (85 °C). The O/W pre-emulsion was homogenized using the probe sonicator at 50% amplitude for 12 min at 85 °C; the nanoemulsion obtained was then cooled in an ice bath. The maximum GEN loading capacity of the SLN was evaluated by observing the macroscopic drug precipitation in the aqueous phase. As reported in the Supplementary Materials Section S1.2, the obtained precipitate was analyzed via high-performance liquid chromatography (HPLC) following the method described in Section 2.5.

The evaluation of the dimensional properties, physical stability, and GEN loading reported in the following paragraphs was performed only on the loaded formulations that were selected as leaders.

### 2.4. Analysis of Particle Size, Polydispersity Index, and Physical Stability

The particle size and the polydispersity index (PDI) of the formulations were determined via Photon Correlation Spectroscopy using a Coulter nanosizer N5 (Beckman–Coulter Inc. Miami, Florida, USA). Each analysis was performed by diluting an exact amount of SLN dispersions (20 µL) with distilled water (3 mL). Samples were analyzed three times (*n* = 3), and the results are expressed as mean ± standard deviation (SD).

The formulations were stored at 4°C, and particle size and PDI were assessed after 30 days to evaluate the physical stability of GEN-loaded SLN (G-SLNa and G-SLNb,) and blank SLN (B-SLNc).

### 2.5. Determination of GEN Loading

To quantify the GEN amount, a modified high-performance liquid chromatographic method (HPLC) was used [27]. For this purpose, 50 µl of formulations (G-SLNa and G-SLNb) were added to methanol (950 µl) to extract the drug. Only one extraction was sufficient. The suspension was vortexed for 30 s and centrifuged at 13,000 rpm for 10 min with a Hettich Mikro 120 centrifuge (Tuttlingen, Germany). After filtration on 0.22 μm polytetrafluoroethylene (PTFE) membrane filters (VWR International, Milan, Italy), the samples were analyzed by HPLC as reported above. GEN loading DL (%) (*n* = 3 ± SD) was calculated according to the following equation:

DL (%) = (amount of GEN in SLN)/(theoretical amount of GEN used) × 100(1)

A Varian HPLC–DAD (Varian IncScientific Instruments, Walnut Creek, CA USA) was used for HPLC analysis, which consisted of two ProStar 210 pumps, a ProStar 410 autosampler, and a DAD Varian 330 detector. The analyses were performed using a C18 column (100 × 4.6 mm, Supelco Ascentist) at 25 °C. The mobile phase, which consisted of acetonitrile and a 25 mM acetic acid solution (48:52 *v*/*v*), was filtered through 0.45 µm nylon membrane filters (Sartorius, Goettingen, Germany) before use and was pumped with a flow rate of 1.2 mL/min at ambient temperature. Detection of the drug was performed at 260 nm with a retention time of about 2.5 min. GEN content was determined using a previously prepared calibration curve (y=290551x−21665.9; R^2^=0.9996), obtained using GEN standard solutions with a concentration ranging from 0.5 to 20 mg/L.

### 2.6. Morphological Analysis and Zeta Potential

The morphologies of G-SLNb and B-SLNc were evaluated via Transmission Electron Microscopy (TEM) using a Philips CM10 (Eindhoven, Netherlands) instrument. For sample preparation, the SLN suspensions were diluted with deionized filtered water (1:10 dilution) and were negatively stained with an osmium tetroxide solution (1.0% *v*/*v*). A drop of stained SLN aqueous suspension was placed on a Formvar-coated copper grid and air-dried before observation.

A 90 Plus instrument (Brookhaven, NY, USA) was used to determine the zeta potentials of G-SLNb and B-SLNc. The temperature and the scattering angle were 25 °C and 90°, respectively. Before measurement, the SLN suspension was diluted in deionized filtered water and the samples were positioned in the electrophoretic cell, where an electric field of approximately 15 V/cm was applied. Each value consisted of the average of 10 readouts.

### 2.7. In Vitro GEN Release Study

The in vitro release study was performed in simulated intestinal fluid (SIF) (USP), which consisted of 50 mM KH_2_PO_4_, 15 mM NaOH, and 1.0% (*w*/*v*) pancreatin (with a pH of 6.8) [28], using a paddle dissolution apparatus (Erweka, Heusenstamm, Germany). The rotational speed and bath temperature were set at 50 rpm and 37 °C, respectively. To evaluate the amount of drug released during formulation, an exact volume of SLN dispersion (G-SLNb) containing 0.6 mg of GEN was transferred into vessels containing 100 mL of dissolution media. At fixed intervals of time (30 min, 1 h, 2 h, 3 h, 4 h, 5 h, and 6 h), 1 mL of medium was withdrawn by replacing the dissolution medium with 1 mL of fresh SIF to maintain sink conditions. The samples were centrifuged for 10 min at 13,000 rpm, filtered with PTFE 0.22 µm pore sized filters, and analyzed using HPLC with the method described above. Each analysis was carried out in triplicate (*n* = 3). The dissolution rate of GEN in SIF (0.6 mg) was evaluated as a comparison.

### 2.8. Evaluation of SLN Characteristics Useful for Intestinal Lymphatic Transport

#### 2.8.1. In Vitro Chylomicron-Like Structure Formation

A preliminary evaluation of in vitro chylomicron-like structure formation was assessed by studying the interactions between the SLN and the lipid components of chylomicrons such as phospholipid and cholesterol. Blank SLN (B-SLNc) (500 μL) and different concentrations of phosphatidylcholine derived from soybeans (Lipoid S100) were added to 20 mL of phosphate-buffered saline (PBS; pH 6.8). A phospholipid concentration equivalent to that of the dispersed lipid (2.0% *w*/*v*) was initially evaluated (Test 1); then, the phosphatidylcholine concentration was doubled (4.0% *w*/*v*) (Test 2). Finally, a further test was performed (Test 3) wherein cholesterol was added in the same concentration to that of the dispersed lipid (2.0% *w*/*v*), maintaining the doubled phospholipid concentration (4.0% *w*/*v*). The dispersions obtained were analyzed to assess possible variations in particle size. Furthermore, the zeta potential values and the TEM images of the samples obtained from Tests 1 and 2 were obtained following the method reported in Section 2.6.

#### 2.8.2. Ex Vivo Uptake Study on Intestinal Mucosa

To evaluate the enterocyte SLN uptake, ex vivo permeation was carried out on the porcine duodenum. The intestine was collected in the slaughterhouse and immediately washed with PBS (pH 6.8) and conserved at 4 °C. The duodenum was cut along the mesenteric line, deprived of the tunica serosa [29], and divided into sections of about 1 cm each. The duodenum was then immediately fixed in 10.0% buffered formalin (time zero) or placed in two cell culture plates (Becton Dickinson Labware, France) containing PBS. The first plate was put into an incubator shaker apparatus (SKI 4 Shaker Pharmaceutics Incubator (Argo Lab, Carpi, Italy)) at 25 °C, while the second plate was maintained at 37 °C into a drying oven (model 400, Memmert, Schwabach, Germany). At pre-established times (1 h, 2 h, 3 h, 4 h, 5 h, and 6 h), the various intestine sections were formalin-fixed, embedded in paraffin wax, sectioned at 3 µm, and stained with hematoxylin and eosin (HE), as previously described [30]. The histopathological evaluation of the intestinal sections, including those at time zero, was carried out under an optical microscope (Nikon Eclipse 80i) in consideration of the different cellular alterations (including villus morphology and enterocyte degeneration) through a composite grading system based on 4 degrees: +1: no alteration; +2 mild degeneration; +3 moderate degeneration; +4 severe degeneration.

Following the histological evaluations that allowed for the identification of the optimal conditions for intestine conservation, the ex vivo permeation was carried out. This experiment was conducted using the fluorescein-labeled SLN (F-SLN), and the solution of free fluorescein in 0.5% *w*/*v* aqueous Tween 80 was prepared as reported in the Supplementary Materials. Alongside this, an untreated intestinal mucosa was used as a control. The intestine sections were placed on a 12-well cell culture plate that had been suitably modified (project INCREASE SARDINIA 2016-17, protocol number 31351, University of Sassari) [31] and previously filled with PBS (pH 6.8). An exact amount of sample (200 µl of F-SLN and fluorescein solution, corresponding to 0.1 mg of fluorescein) was placed on the mucosal surface of the intestine segments, and the plate was positioned in the incubator shaker apparatus at 25 °C and 150 rpm. Immediately after nanoparticle deposition, and after 1 h, 2 h, and 3 h, tissues were collected, washed thrice with PBS, formalin-fixed, paraffin-embedded, stained with HE, and histologically evaluated. Additional serial sections (each 4 μm in thickness) were mounted on positively charged slides (Superfrost, Fisher Scientific, Rodano MI, Italy), deparaffinated, and incubated for 1 min with Hoechst 3334 (5 μg/mL; Invitrogen). Fluorescence was observed using a filter-free confocal laser scanning microscope (Leica Microsystems, Wetzlar, Germany), and the LAS AF Lite 3.2 (Leica Microsystems) software was used for image processing [32].

#### 2.8.3. In Vitro Cellular Uptake Study

To evaluate cellular uptake, Caco-2 cells were treated with F-SLN and free fluorescein solution in 0.5% *w*/*v* aqueous Tween 80. Caco-2 cells were seeded in 6-well plates at a density of 1.5 × 10^6^ cells/well, incubated overnight, and exposed to F-SLN and free fluorescein (20 μM) for 30 min, 3 h, and 24 h. After the incubation time, the cells were washed three times in PBS and suspended in DMEM/F12 0.25% *w*/*w* of Trypan Blue. The uptake of the fluorescent nanoparticles into the Caco-2 cells was measured using a FACSCANTO flow cytometer (Becton & Dickinson, Franklin Lakes, NJ, USA), and the experimental data were analyzed with DIVA 6.3 software (BD Bioscience). The pH values of the Caco-2 cell culture medium were measured before, during, and after treatment with SLN using the Orion STAR SERIES pH Benchtop (Thermo Electron Corporation, Beverly, MA, USA) pH meter. The pH of the cells was 7.4, and no significant differences were observed. In all experiments, pH was measured three times before, during, and after treatment to obtain statistical values.

### 2.9. Statistical Analysis

Statistical analysis was done with Graph Pad Prism 8.0 software (GraphPad Software Inc. San Diego, CA, USA). The analysis of variance (one-way ANOVA) was used to analyze the data, followed by a Tukey’s multiple comparison test. Differences were considered significant for *p* < 0.05.

## 3. Results and Discussions

### 3.1. Preparation of GEN-Loaded SLN

Based on the preliminary results reported in the supplementary files, the suitable technological parameters for the preparation of SLN proper to be loaded with GEN were identified; the sample B-SLNc (Table 1 of Supplementary Material) was selected as an unloaded leader formulation for the GEN loading process.

The surfactant concentration has an important role in the quality of SLN dispersion [33]. In our work, the best emulsifier concentration was found to be 0.5% (*w*/*v*) because B-SLN presented the best results for intestinal lymphatic transport, in terms of particle size (20–500 nm) [3]. On the contrary, the sample with 0.3% surfactant concentration, although characterized by a lower concentration of surfactant, showed larger values of mean diameter (>500 nm). These results are in good agreement with those previously described by Abdelbary and Fahmy [34]. The reduction in particle size that comes from increasing the surfactant concentration is probably due to the decrease in interfacial tension among the lipid and aqueous phases. This leads to the development of smaller emulsion droplets that, after the cooling process, resulted in small nanoparticles. Moreover, high emulsifier amounts may create a steric barrier on the SLN surface, which protects the nanoparticles from coagulation [35,36].

Although several authors have reported that increasing the lipid concentration resulted in larger particle size and reduced suspension homogeneity [33,37], when the C amount was reduced for the formulation with 0.5% of Tween 80, no significant differences in terms of mean diameter and PDI were observed; therefore, this parameter was not modified. Concerning the type of emulsion used for SLN preparation, similar consideration should be due, and thus the O/W emulsion was chosen. B-SLN exhibited the best values for particle size if sonicated for 12 min; therefore, the pre-emulsion homogenization was carried out with the probe sonicator. Therefore, these results permitted us to identify the suitable parameters for GEN-loaded SLN: Tween 80 0.5% (*w*/*v*), C 2.0% (*w*/*v*), O/W emulsion, and homogenization with a probe sonicator for 12 min.

### 3.2. GEN Loading Capability

The GEN loading capability of SLN was evaluated by testing different GEN concentrations within a range of 0.02–0.06% (*w*/*v*). From the results, it was observed that the formulation was able to incorporate an amount of GEN corresponding to 0.02–0.03% (*w*/*v*) in case of G-SLNa and G-SLNb, respectively. In the samples containing higher concentrations of GEN (0.04 and 0.06% *w*/*v* for G-SLNc and G-SLNd, respectively), the formation of a GEN precipitate was observed. The drug is water-insoluble; therefore, this precipitate suggests the lack of GEN incorporation into SLN. Furthermore, the HPLC analysis of the precipitate showed the characteristic peak of GEN, as reported in section S2.2 of the Supplementary Material.

### 3.3. Analysis of Particle Size, Polydispersity Index, and Physical Stability

**Figure 1:**
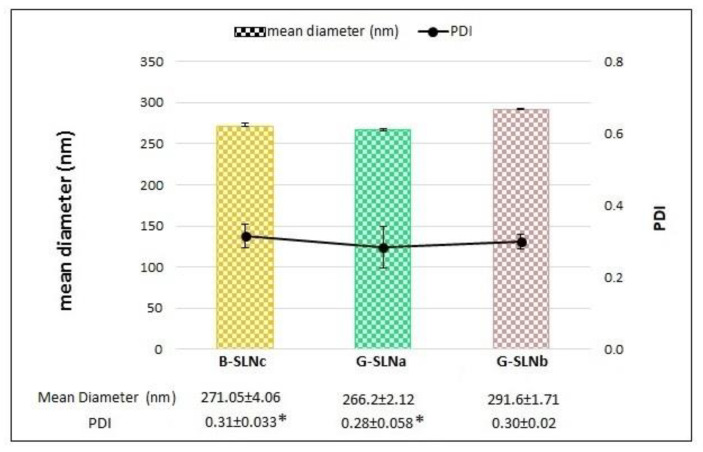
Particle size and PDI of loaded and unloaded SLN. PDI, *p* < 0.05: *= B-SLNc vs G-SLNa. Results are reported as mean ± standard deviation (SD).

Figure 1 shows the particle size and the PDI of the loaded formulations (G-SLNa and G-SLNb) compared to those of the unloaded SLN (B-SLNc). According to de Zampieri et al. [27], GEN loading did not lead to significant differences in particle size (*p* > 0.05). In contrast, a GEN concentration of 0.02% (*w*/*v*) increased the homogeneity of SLN (*p* < 0.05), whereas G-SLNb displayed similar PDI values to those found to G-SLNc (*p* > 0.05).

**Figure 2:**
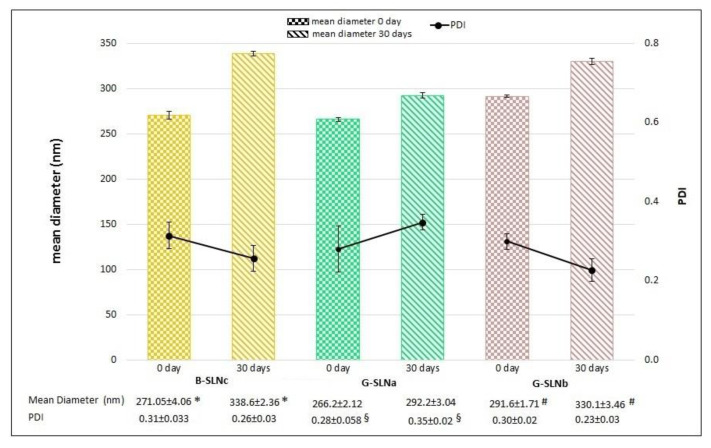
Physical stability of unloaded and GEN-loaded SLN after 30 days. *p* < 0.05: *B-SLNc at 0 days vs B-SLNc at 30 days; ^#^G-SLNb at 0 days vs G-SLNb at 30 days; ^§^ G-SLNa at 0 days vs G-SLNa at 30 days.

The stability trends of GEN-loaded SLN and blank SLN in term of particle size and PDI over time are reported in Figure 2. B-SLNc and G-SLNb exhibited an increase in particle size after 30 days (*p* < 0.05); in contrast, no significant changes in terms of PDI occurred over time (*p* > 0.05), indicating a good homogeneity of SLN dispersions. The sample G-SLNa showed an opposite trend: particle size did not change from day 0 to day 30 (*p* > 0.05); however, the suspension homogeneity was reduced (*p* < 0.05). This was likely because, at the time of preparation, G-SLNa consisted of only one nanoparticle population with a mean diameter of about 260 nm while, after 30 days, the dispersion was characterized by some populations of smaller SLN. As a result, there was not a variation in terms of particle size, but instead a reduction in homogeneity due to the presence of more SLN populations.

### 3.4. Morphological Analysis and Zeta Potential

**Figure 3:**
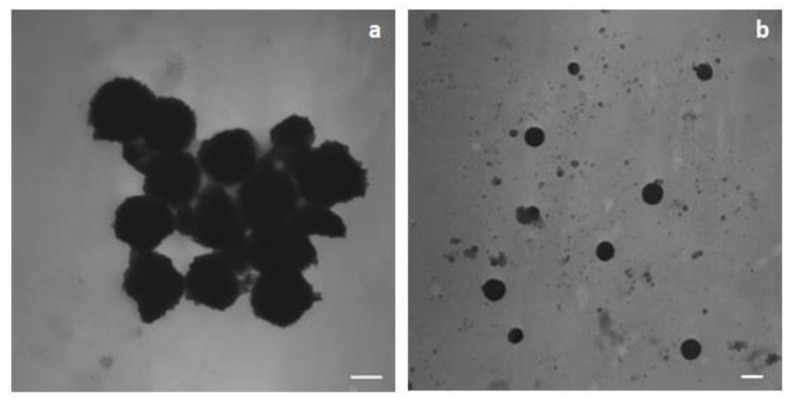
TEM image of B-SLNc (**a**) and G-SLNb (**b**). (**a**) Magnification 39000; scale bar 150 nm; (**b**) magnification 15500, scale bar 300 nm.

B-SLNc were roundish particles and partially aggregated (Figure 3a); G-SLNb were regular and spherical, and no aggregation was observed (Figure 3b).

The mean zeta potential values of B-SLNc and G-SLNb were −16.35 ± 2.13 and −18.45 ± 1.98 mV, respectively. The introduction of GEN in the nanoparticles led to a slight increase in their zeta potential value (*p* < 0.05). This increase of superficial negative charge could be due to a small amount of GEN on the SLN surface [27]. The surface nanoparticle charge is an important factor in lymphatic uptake. Indeed, it has been reported that negatively charged lipid-based nanosystems showed a higher lymphatic uptake if compared to positively or neutrally charged particles [3,8].

### 3.5. Determination of GEN Loading

The GEN content was evaluated using HPLC to confirm the efficacy of the preparation method and to evaluate the effective drug content as compared to that of the theoretical model. The percentage of GEN loading determined for both formulations was similar to that of the theoretical model (100 ± 6.14 for G-SLNa and 97.1 ± 0.76 for G-SLNb), regardless of the quantity of drug loaded, indicating a good capacity for formulations to incorporate GEN. Similar results were found in our previous work, where the high loading ability of other lipid nanocarriers was demonstrated [15].

### 3.6. In Vitro Drug Release Studies

**Figure 4:**
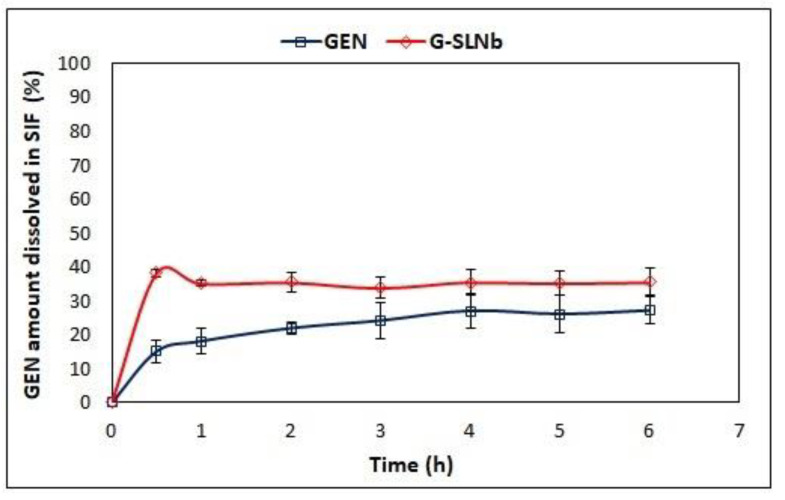
In vitro GEN release profile from G-SLNb and the dissolution profile of GEN. Data are reported as mean ± SD (*n* = 3).

The results in Figure 4 showed that 15% of GEN dissolved in SIF within the first 30 min, then an increase in the dissolution rate was observed and 27% of the drug was found in the dissolution media after 6 h. Regarding SLN formulation, the release profile showed an initial burst release of 34% of GEN during the first 30 min; as a result, the GEN amount did not change during this time. This may be attributed to the presence of a small amount of GEN in the external matrix of SLN, as previously hypothesized based on the zeta potential results. This is in disagreement with the previous work of Kim et al., which reported that GEN-loaded SLN rapidly release GEN, which could then be absorbed into the upper part of the intestine and could undergo first-pass metabolism in the liver [38]. The difference in this behaviour could be attributed to the lipid and surfactant composition affecting GEN entrapment.

### 3.7. Evaluation of SLN Characteristics Useful for Intestinal Lymphatic Transport

#### 3.7.1. In Vitro Chylomicron-Like Structure Formation

The most common approaches for the evaluation of intestinal lymphatic transport are in vivo studies; for example, the chylomicron flow blocking approach is widely used [39,40]. On the contrary, in vitro methods are not still reported, even if this type of experiment can provide important information. The SLN prepared in this work should undergo the physiological lipid absorption process; therefore, once within enterocytes, the interaction with phospholipids and cholesterol would allow for chylomicron formation [41], which would subsequently be specifically absorbed by intestinal lymphatic vessels. Chylomicrons are lipoprotein particles that consist of a lipid core of triglycerides, cholesterol, and cholesterol esters, surrounded by a coat of phospholipids, apolipoprotein, and cholesterol [42]. Then, to have preliminary knowledge regarding the intestinal lymphatic transport of SLN, the in vitro chylomicron-like structure formation was assessed by studying the interaction of SLN with phospholipids (Lipoid S100) and cholesterol. Hence, phosphatidylcholine and cholesterol were added to a blank SLN dispersion, and the eventual variations in particle size were evaluated. Chylomicrons are large lipoproteins, with diameters ranging from 100 to 1000 nm [43]; therefore, if SLN interacted with chylomicron components, an increase in particle size would be expected.

The mean diameter of B-SLNc (271 nm) increased to 390 nm by adding the phospholipid in a concentration comparable to that of C (Test 1), while when the phosphatidylcholine was doubled (Test 2), systems with a mean diameter of 500 nm were obtained. By combining the addition of phospholipid and cholesterol, the mean diameter was 389 nm, a value comparable to that obtained in the first test. Based on these preliminary results, the interaction of SLN with the chylomicron components could be hypothesized, and this interaction was more evident when the phospholipid concentration was increased. Along with this, TEM images and the zeta potential values of the sample obtained with Tests 1 and 2 (the systems obtained with Test 3 were not analyzed because the particle size was equal to that of Test 1) were determined.

**Figure 5:**
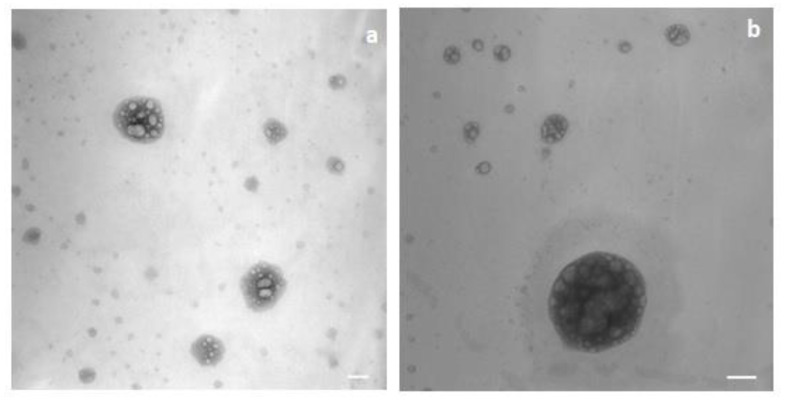
TEM image of the sample obtained from Test 1 (**a**) and Test 2 (**b**) (magnification 39,000, scale bar 150 nm).

In Figure 5, the TEM images of the sample obtained with Test 1 (Figure 5a) and Test 2 (Figure 5b) are reported.

TEM images of the sample obtained with Tests 1 and 2 showed the formation of chylomicron-like structure vesicles with a different morphology than those of SLN. The formation of multinucleated vesicles typical of solid lipids in the presence of phospholipids and Tween was observed [44].

Chylomicron-like structure vesicles have a negative surface charge of −37.32 ± 0.71 mV and −42.92 ± 1.71 mV for the samples obtained with Test 1 and 2, respectively. The increase in negative charge compared to that of B-SLNc (−16.35 ± 2.13 mV) was imparted by the phospholipid because of the presence of phosphatidic acid in the Lipid S100 [45].

After formation, the chylomicrons reach the bloodstream via the lymphatic vessels and, once circulating with the blood, are degraded by lipoprotein lipases [24]. In this way, the SLN matrix will destroyed and the GEN will be released.

#### 3.7.2. Ex Vivo Uptake Study on Intestinal Mucosa

**Figure 6:**
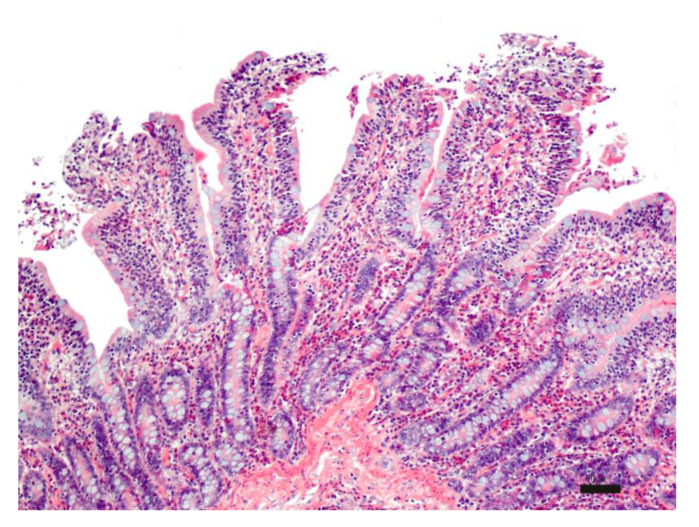
Histological evaluation of intestinal tissue at time zero: moderate, diffuse chronic lymphoplasmacellular and eosinophilic enteritis. HE; bar: 50 µm.

During the first part of the experiment, the morphology of the intestinal tissue exposed to different temperatures (25 °C and 37 °C), and experimental times (from 0 to 6 h) was evaluated. The time zero evaluation of the intestinal sections was characterized by a moderate inflammatory infiltrate expanding the lamina propria, composed mostly of lymphocytes and plasmacells and a lesser number of eosinophils and macrophages. Villi were blunted with apical fusion and lined by swollen epithelial cells with granular amphophilic cytoplasm and basal hyperchromatic nuclei. The moderate inflammatory infiltrate associated with villi blunting likely led to an alteration in the enterocyte absorption capacity and a probable reduction in SLN uptake (Figure 6).

**Figure 7:**
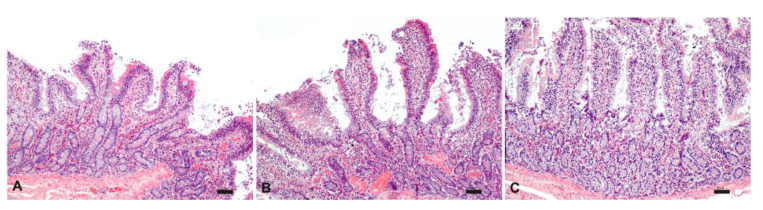
Histological evaluation of intestinal tissue stored at 25°C at 1 h (**A**), 2 h (**B**) and 3 h (**C**). HE; bar: 50 µm.

The histological analysis carried out on intestinal samples stored at 25°C was characterized by tissue and cell morphology, with similar features described at time zero and up to 3 h. Starting from 4 h, severe epithelial degeneration with multiple weakly eosinophilic intracytoplasmic vacuoles of varying sizes, multifocal plasma membrane discontinuities, and nuclear pyknosis were observed. Furthermore, the lamina propria, in addition to the described inflammatory infiltrate, was characterized by widespread edema and by numerous extravasated red blood cells (Figure 7).

**Figure 8:**
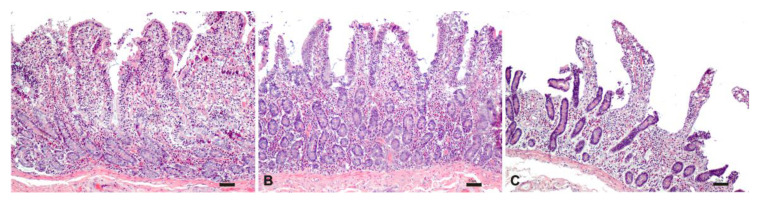
Histological evaluation of intestinal tissue stored at 37°C at 1 h (**A**), 2 h (**B**) and 3 h (**C**). HE; bar: 50 µm.

Unlike what was observed in the samples stored at room temperature, the samples stored at 37 °C were histologically characterized by diffuse morphological alterations starting from 2 h, indicating a lower stability of the intestine at higher temperatures (Figure 8).

Based on the histological evaluation, the tissue and cell morphology of intestinal mucosa retain similar features as those described at time zero for about 3 h by being kept at 25 °C in PBS. In general, when performing ex vivo tests, the aspect related to the conservation and degenerative processes is considered marginal. However, alterations of the intestinal mucosa that occur over time, as well as the presence of inflammation as a “background lesion”, could invalidate the success of these tests; therefore, it would be prudent to carry out a preliminary histological evaluation in order to consider any intrinsic “bias”.

For the ex vivo permeation test, the intestine was treated with fluorescein-labeled SLN (F-SLN) and the fluorescein solution; an untreated intestinal mucosa was also used as a control. In this case, histological analysis of the untreated intestinal mucosa at time zero also revealed the presence of a moderate inflammatory infiltrate.

**Figure 9:**
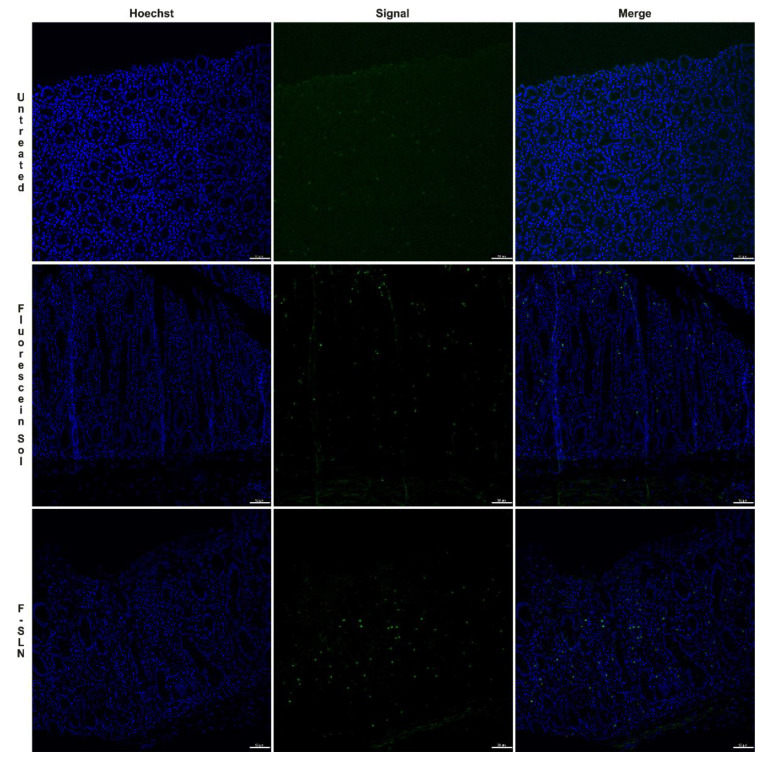
Ex vivo permeation test on intestinal tissue after 1 h: untreated intestinal mucosa, intestinal mucosa treated with free fluorescein solution, and intestinal mucosa treated with F-SLN. Signal (green) / Hoechst 33342 nuclei (blue). Bar: 50 µm.

Diffuse signals were observed in the samples analyzed at 1 h both in the mucosa treated with the fluorescein solution and in that treated with F-SLN, probably related to the uptake of enterocytes and/or inflammatory cells present in the lamina propria of both free fluorescein and labeled SLN (Figure 9).

**Figure 10:**
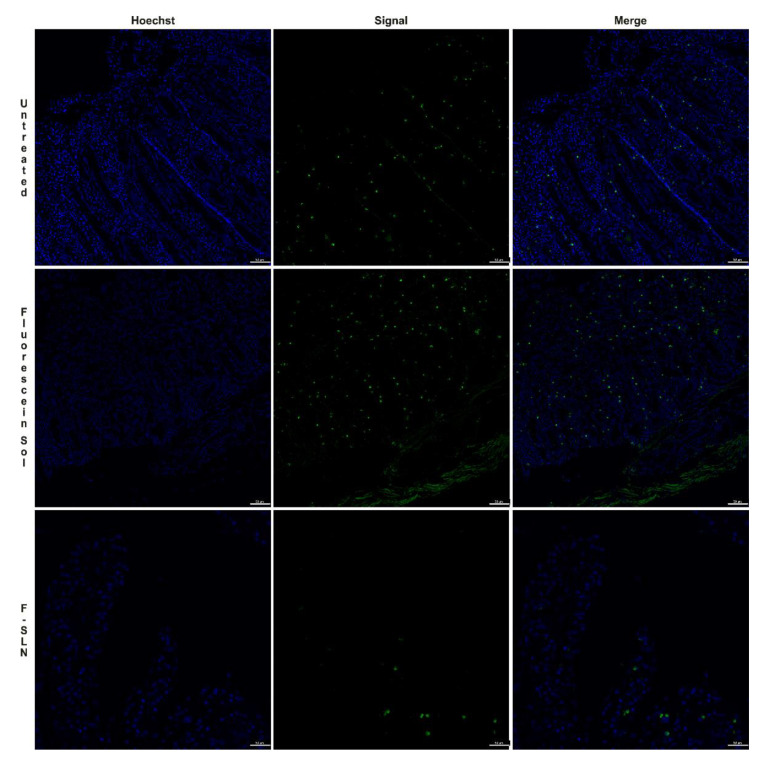
Ex vivo permeation test on intestinal tissue after 2 h: untreated intestinal mucosa, intestinal mucosa treated with free fluorescein solution, intestinal mucosa treated with F-SLN. Signals (green) / Hoechst 33342 nuclei (blue). Bar: 50 µm.

Fluorescence signals were observed in the samples analyzed after 2 h both in the untreated control mucosa and in the free fluorescein and SLN treated tissues (Figure 10). Similar results were also observed at 3 h. Therefore, the detection of fluorescence signals in the untreated mucosa could be related to the high inflammatory infiltrate consisting, in addition to lymphocytes and plasmacells, of macrophage cells capable of engulfing cellular debris or degradation products such as lipofuscins, which by their intrinsic nature could emit fluorescence [46].

#### 3.7.3. In Vitro Cellular Uptake Study

**Figure 11:**
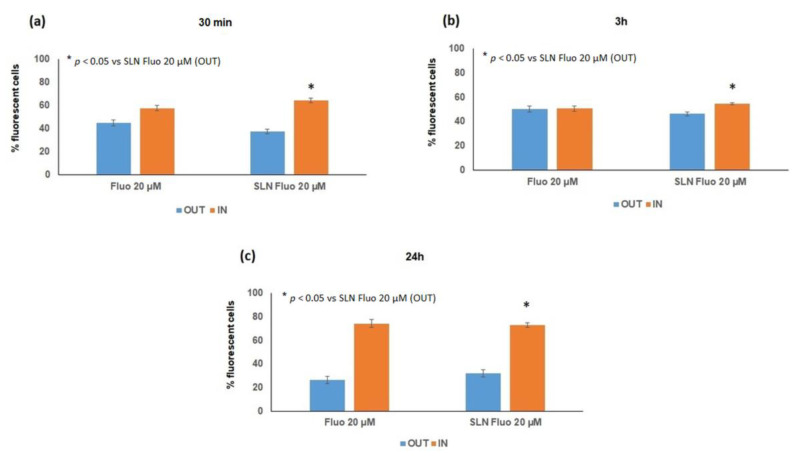
Effects of in vitro cellular uptake on Caco-2 cells at 30 min (**a**), 3 h (**b**), and 24 h (**c**).

To confirm the results obtained with ex vivo intestinal uptake, flow cytometry analysis was used to verify the internalization efficiency of F-SLN (20 uM) in Caco-2 cells. The amount of F-SLN measured inside and outside of the cells was represented as a percentage of the fluorescent cells. In Figure 11, the amount of F-SLN internalized (IN) (64% at 30 min, 55% at 3 h, and 73% at 24 h) was always higher than the percentage of nanoparticles outside of the cells (OUT) (38% at 30 min, 46% at 3h, and 32% at 24 h). These results suggest the internalization of SLN inside the cells.

## 4. Conclusions

SLNs, with particle sizes and surface charges suitable for the mucosal delivery of GEN, were successfully obtained with the hot homogenization process. The SLN showed a high loading ability and good physical stability during the time of the experiment. In this work, we demonstrated that GEN-loaded SLN with suitable dimensional characteristics are internalized by intestinal cells and, due to their lipid composition, they can lead to the formation of chylomicron-like structure vesicles. Thus, these SLN could preferentially reach the intestinal lymphatic vessels, and GEN first-pass metabolism could be avoided. This will be confirmed with further in vivo studies.

## Figures and Tables

**Table 1:** Composition of Genistein (GEN)-loaded solid lipid nanoparticles (SLN).

Sample	Compritol 888 ATO (% *w*/*v*) ^§^	Tween 80 (% *w*/*v*) ^§^	Genistein (% *w*/*v*) ^§^
G-SLN a	2	0.5	0.02
G-SLN b	2	0.5	0.03
G-SLN c	2	0.5	0.04
G-SLN d	2	0.5	0.06

^§^ The percentages are referred to as the final SLN dispersion.

## Data Availability

The data presented in this study are available on request from the corresponding author.

## Supplementary Materials

The following are available online at https://www.mdpi.com/1999-4923/13/2/267/s1: Figure S1: Formulative parameters varied during preliminary studies. Figure S2: Effect of Tween 80 concentration on size and PDI of SLN dispersions. Figure S3: Effect of homogenization with Ultra-Turrax and with a sonicator probe on the mean particle size and PDI. Figure S4: HPLC diode array detection of the precipitate isolated by GEN-loaded SLN with 0.04 and 0.06% (*w*/*v*) of the drug. The peak of GEN is evident. Figure S5: Evaluation of fluorescein incorporation into SLN. Table S1: Concentrations of Tween 80 tested during preliminary studies. Table S2: Composition and homogenization technique of all formulations prepared during preliminary studies. Table S3: Particle size and PDI of formulations containing 1 and 2% (*w*/*v*) of C. Table S4: Fluorescence stability of F-SLN during various times (1 day, 2 days, 5 days, and 10 days).

## Artwork

Part of the graphical abstract was created with BioRender.com.
