# Supplementary material for: Transmucosal Solid Lipid Nanoparticles to Improve Genistein Absorption via Intestinal Lymphatic Transport
Source: Pharmaceutics. 2021 Feb 16;13(2):267. doi: 10.3390/pharmaceutics13020267 (PMC7920073; doi:10.3390/pharmaceutics13020267)
Supplement: Supplementary file 1 [file pharmaceutics-13-00267-s001.pdf]

pharmaceutics

Supplementary Material: Transmucosal Solid Lipid Nanoparticles to Improve Genistein Absorption via Intestinal Lymphatic Transport

Antonella Obinu, Giovanni Pietro Burrai, Roberta Cavalli, Grazia Galleri, Rossana Migheli, Elisabetta Antuofermo, Giovanna Rassu, Elisabetta Gavini *, Paolo Giunchedi

S1. Materials and Methods

S1.1. Formulative studies

The first part of the work has been characterized by preliminary studies to identify a leader formulation having suitable technological features for the GEN loading process and the intestinal lymphatic transport of the drug. These studies were carried out on unloaded SLN and during this phase, some formulation parameters were modified and the suitability of the obtained SLN dispersions was evaluated by determining the particle size and the PDI as described in paragraph 2.4 of the main manuscript.

An initial dispersion of blank SLN (B-SLN) was prepared by a hot homogenization process, as reported by Muller et al. [1] and Mehnert and Mader [2]. Briefly, C (2.0% w/v) was weighed and melted at 80°C. Tween 80 was dissolved in distilled water to have a final concentration of 0.5% (w/v) and heated at 85°C. The homogenous lipid phase was added to the hot aqueous surfactant solution under magnetic stirring to obtain an oil/water (O/W) pre-emulsion. The final emulsion was obtained by homogenization using an Ultra Turrax T-25 digital (IKA, Staufen, Germany) for 5 min at 14,600 rpm maintaining the temperature at 85°C to avoid solidification of lipid. The nanoemulsion obtained was then cooled in an ice bath for 20 min under constant magnetic agitation to achieve SLN dispersion in water.

Based on the results obtained from the dimensional analysis of this first blank SLN dispersion, several technological parameters, such as surfactant and lipid concentration, type of emulsion, and technique of homogenization were varied.

The influence of the surfactant amount on nanoparticle size and homogeneity was evaluated by testing further three Tween 80 concentrations: 0.3%, 0.75%, and 1.0% (w/v) (B-SLN0.3, B-SLN0.75, and B-SLN1 respectively). The concentration of dispersed lipid (2.0% w/v) was maintained constant. All the surfactant concentrations tested during preliminary studies are reported in Table S1.

Table S1. Concentrations of Tween 80 tested during preliminary studies.

Sample	Compritol 888 ATO (% w/v) §	Tween 80 (% w/v) §
B-SLN0.3	2.0	0.3
B-SLN	2.0	0.5
B-SLN0.75	2.0	0.75
B-SLN1	2.0	1.0

§ Lipid and surfactant percentage in the final SLN dispersion.

Based on the results obtained, B-SLN and B-SLN0.3 were selected for further experiments.

At first, the reduction of lipid concentration from 2.0% to 1.0% (w/v) was evaluated and the two samples B-SLNb and B-SLN0.3b were prepared. Then, the effect of emulsion

typology was tested. For this purpose, the hot aqueous solution of Tween 80 (0.3 or 0.5% w/v) was added to the molten lipid (2.0% w/v) and the pre-emulsion obtained was homogenized as described above. The formulations B-SLNW/O and B-SLN0.3W/O were obtained. To evaluate the effect of the homogenization technique on SLN features, the samples B-SLN, B-SLN0.3, B-SLNW/O and B-SLN0.3W/O were subjected to a homogenization process using a probe sonicator Vibra Cell, VC 50 (Sonics and Materials, Danbury, USA) at 50% amplitude instead of the Ultra Turrax. During this phase, different sonication times were tested: 6, 9, 12, and 15 min. The formulations B-SLNc, B-SLN0.3c, B-SLNW/Oc and B-SLN0.3W/Oc were obtained.

The technological parameters varied during the preliminary studies are reported in Figure S1, while in Table S2 are summarized the composition and the homogenization technique of all formulations prepared.

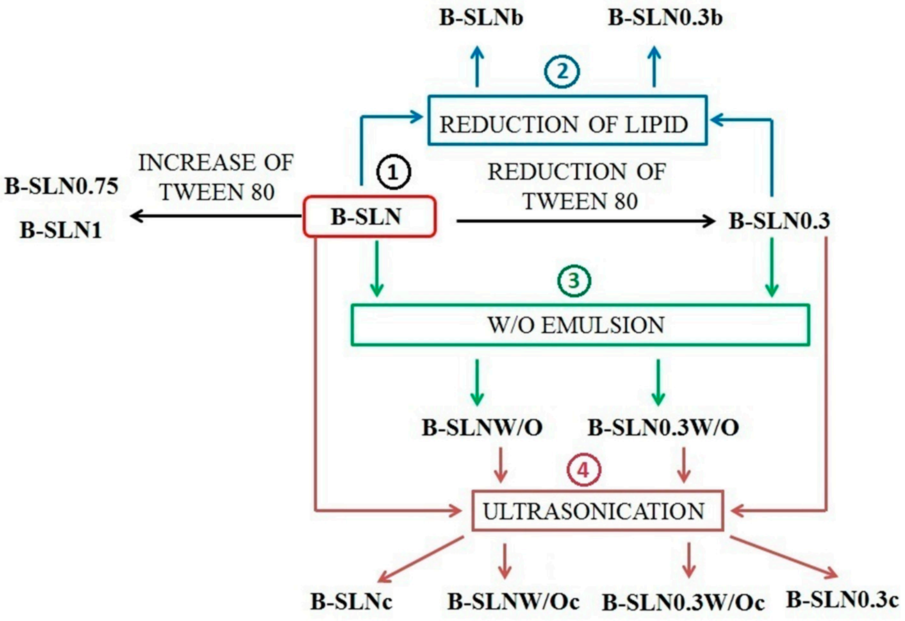

Figure S1. Formulative parameters varied during preliminary studies.

Table S2. Composition and homogenization technique of all formulations prepared during preliminary studies.

Sample	Compritol® 888 ATO (% w/v) §	Tween 80 (% w/v) §	Type of emulsion	Technique of homogenization
B-SLN	2.0	0.5	O/W	Ultra-Turrax
B-SLN0.3	2.0	0.3	O/W	Ultra-Turrax
B-SLN0.75	2.0	0.75	O/W	Ultra-Turrax
B-SLN1	2.0	1.0	O/W	Ultra-Turrax
B-SLNb	1.0	0.5	O/W	Ultra-Turrax
B-SLN0.3b	1.0	0.3	O/W	Ultra-Turrax
B-SLNW/O	2.0	0.5	W/O	Ultra-Turrax
B-SLN0.3W/O	2.0	0.3	W/O	Ultra-Turrax
B-SLNc	2.0	0.5	O/W	Ultrasonication
B-SLNW/Oc	2.0	0.5	W/O	Ultrasonication
B-SLN0.3c	2.0	0.3	O/W	Ultrasonication
B-SLN0.3W/Oc	2.0	0.3	W/O	Ultrasonication

§ Lipid and surfactant percentage in the final SLN dispersion

S1.2 Preparation of GEN-loaded SLN

The SLN maximum GEN loading capability was assessed by evaluating the macroscopic drug precipitation in the aqueous phase. When a precipitate was obtained, it was collected, dissolved in methanol, and analyzed by HPLC following the method reported in section 2.5 of the main manuscript.

S1.3. Preparation of fluorescein-labeled SLN

Fluorescein labeled SLN (F-SLN) were prepared to evaluate the ex vivo and in vitro uptake of nanoparticles. The SLN were obtained by using the hot homogenization method described in paragraph 2.3. Briefly, 10 mg of fluorescein were solubilized in 3 ml of acetone, this solution was added drop by drop to the molten lipid (C, 2.0% w/v) and the lipid phase was added to 20 ml of a 0.5% (w/v) Tween 80 aqueous solution previously heated. The O/W pre-emulsion obtained was homogenized with the probe sonicator (50% amplitude, temperature at 85°C) for 12 min and the dispersion was cooled in an ice bath under magnetic stirring.

The F-SLN were characterized in terms of dimensional properties and physical stability (0, 5, and 10 days) and the fluorescein incorporation was assessed by observing its precipitation in the aqueous media of SLN dispersion. For this purpose, other two formulations were prepared: in the first sample, 10 mg of fluorescein were added to preformed blank SLN under magnetic stirring, while in the second formulation the solid dye was added to molten lipid. The fluorescence stability of F-SLN during the time was evaluated using an RF-6000 spectrofluorometer (Shimadzu, Kyoto, Japan) with an excitation wavelength of 460 nm. Emission spectra were recorded from 400 to 900 nm wavelength range at 0, 1, 5, and 10 days.

The uptake tests were also carried out using a solution of free fluorescein in 0.5% w/v aqueous Tween 80. For the preparation of the fluorescein solution, 10 mg of dye were solubilized in 3 ml of acetone and added to 20 ml of an aqueous solution of Tween 80. To remove the organic solvent, the obtained solution was stirred for 1h at room temperature.

S2. Results and discussions

S2.1. Formulative studies

Preliminary studies were carried out to evaluate how the variation of several formulative parameters influenced the technological features of obtained SLN, particularly in terms of particle size and homogeneity. Using the hot homogenization process, a first blank SLN dispersion (B-SLN) was obtained and this formulation was used as a reference to perform subsequent studies. The particle size and the PDI of B-SLN were 343.3±1.59 and 0.452±0.023, respectively.

In the following paragraphs are discussed the various parameters modified and their effect on prepared SLN.

S2.1.1 Surfactant concentration

Figure S2 shows the mean particle size and PDI of SLN dispersions containing the different Tween 80 concentrations. The mean diameter of SLN ranged from 300 to 500 nm and was influenced by the concentration of Tween 80. Particle size increased with decreasing surfactant concentration to 0.3% (w/v) ($p<0.05$), while no statistical differences were observed by using 0.5%, 0.75% and 1.0% (w/v) Tween 80 concentration ($p>0.05$). On the contrary, no obvious change in PDI occurred when the surfactant concentration has been changed ($p>0.05$).

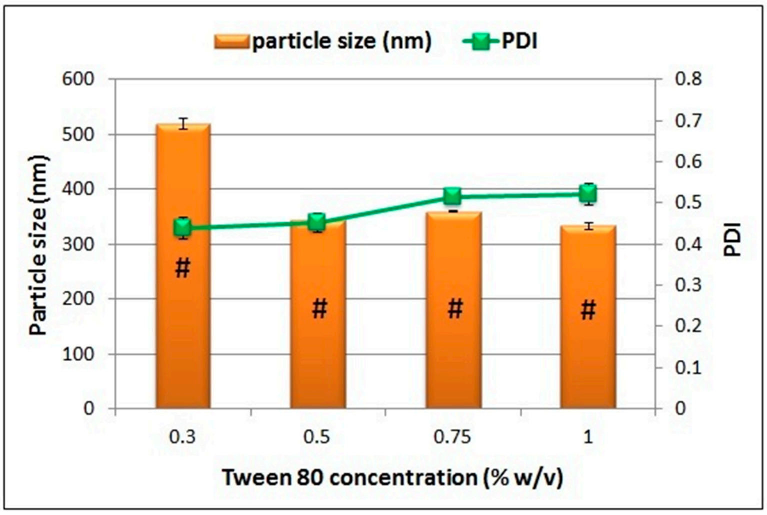

Figure S2. Effect of Tween 80 concentration on size and PDI of SLN dispersions. Particle size, $p<0.05$: #= 0.3% vs 0.5%, 0.75% and 1.0%.

Based on these preliminary results, the formulations B-SLN and B-SLN0.3 were chosen for further variations in process parameters. In particular, the sample B-SLN, which consisted of small particles (about 350 nm) showed a particle size suitable for intestinal lymphatic transport [3]. The formulation B-SLN0.3, although with a larger diameter (>500 nm), was characterized by a low amount of surfactant. The influence of surfactant on cell viability was reported [4,5], so it may be advantageous to use the minimum concentration required to obtain an SLN dispersion.

S2.1.2 Concentration of lipid

Vitorino and colleagues [6] reported that lipid concentration influences the average diameter of SLN. High lipid content leads to a more viscous dispersed phase, resulting in the worst homogenization efficiency. Based on this consideration, the effect of lipid concentration on dimensional characteristics of SLN was investigated by changing the C amount. The particle size and PDI of the nanoparticle dispersions containing 1.0% and 2.0%% (w/v) of lipid concentration are reported in Table S3.

The SLN did not show a significant change in particle size ($p>0.05$) when the lipid amount was reduced. On the other hand, the decrease in C concentration led to an increase in PDI values for the samples containing 0.3% (w/v) of Tween 80 ($p<0.05$); on the contrary, no statistical differences were observed in formulations with 0.5% of surfactant ($p>0.05$).

Table S3. particle size and PDI of formulations containing 1 and 2% (w/v) of C. $p<0.05$ *= B-SLN0.3 vs B-SLN0.3b.

Sample	Lipid concentration (% w/v)	Mean Diameter (nm±SD)	PDI
B-SLN	2	343.3±1.59	0.452±0.023
B-SLNb	1	395±5.0	0.534±0.028
B-SLN0.3	2	520.6±9.73	0.440±0.026*
B-SLN0.3b	1	476.4±22.27	0.603±0.013*

S2.1.3 Type of emulsion

As reported by Makwana et al. [7], SLN with a particle size less than 300 nm can be obtained by formulating a water/oil (W/O) pre-emulsion and subjecting it to a homogenization process. The previous formulations were obtained using an O/W emulsion, therefore, a further change introduced in the formulation parameters was the type of emulsion used to prepare the SLN. Two other nanoemulsions, obtained with a W/O emulsion, were prepared: B-SLN0.3W/O and B-SLNW/O. The comparison between B-SLN0.3 and B-SLN0.3W/O revealed that the modification introduced caused a significant reduction in particle size ($p<0.05$) (520.6±9.73 and 438.2±10.85 nm respectively), whereas B-SLN and B-SLNW/O diameters were not statistically different ($p>0.05$). In terms of PDI, no significant changes occurred ($p>0.05$) among the SLN obtained with the O/W emulsion and the formulations prepared with the W/O emulsion.

S2.1.4 Mode and time of homogenization

During preliminary studies, the effect of homogenization with Ultra-Turrax or with a probe sonicator, on particle size and PDI, was investigated (Figure S3). In the graphics reported below was also shown the trend of mean diameter and PDI by changing the sonication time.

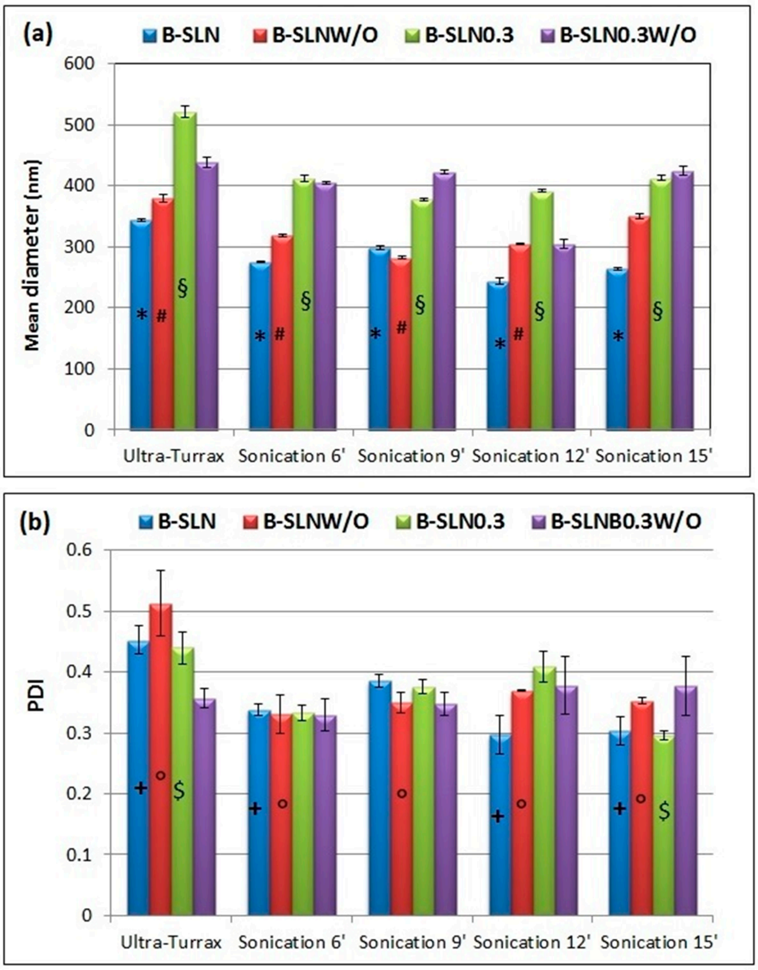

Figure S3. Effect of homogenization with Ultra-Turrax and with sonicator probe on the mean particle size (a) and PDI (b). p<0.05: B-SLN *= Ultra-Turrax vs sonication for 6, 9, 12 and 15 min., += Ultra-Turrax vs sonication for 6, 12 and 15 min; B-SLN0.3 §= Ultra-Turrax vs sonication for 6, 9, 12 and 15 min, $= Ultra-Turrax vs sonication for 15 min; B-SLNW/O #= Ultra-Turrax vs sonication for 6, 9, and 12 min, °= Ultra-Turrax vs sonication for 6, 9, 12 and 15 min.

Concerning SLN prepared by an O/W emulsion (B-SLN and B-SLN0.3), the ultrasonic probe determined a decrease of mean diameter ($p<0.05$). Furthermore, the PDI of B-SLN exhibited a decrease within the first six min of sonication ($p<0.05$), reaching the lowest value after 12 min (0.297±0.013). On the contrary, for the formulation B-SLN0.3 were needed 15 min to have a more homogeneous dispersion than that achieved by Ultra-Turrax (PDI with Ultra-Turrax 0.440±0.026 and with sonicator probe 0.269±0.001). As regards the SLN dispersions prepared with the W/O emulsion the results were different. Particularly, in the case of B-SLNW/O the mean diameter decreased after 6, 9 and 12 min of sonication ($p<0.05$), while no statistical differences were found at 15 min ($p>0.05$), indicating a possible re-aggregation of nanoparticles; besides, the sonicated dispersions were always more homogeneous than the reference one ($p<0.05$). For B-SLN0.3W/O, the sonicator probe did not lead to any dimensional difference compared to the sample homogenized by Ultra-Turrax ($p>0.05$); the same results were also obtained in terms of PDI.

From figure 2 it can also be seen that the sonicator probe led to reduced particle size in both formulations characterized by a Tween 80 concentration of 0.5% (w/v) (B-SLN and

B-SLNW/O). The only exception was the dispersion B-SLNW/O sonicated for 15 min (particle size 349.8±3.25 nm). Regardless of emulsion types, after the homogenization with the sonicator probe, the values of PDI were considerably reduced ($p<0.05$), except for the sample B-SLN following 9 min of sonication ($p>0.05$). Conversely, the emulsion type influenced the homogeneity of the dispersions with the surfactant concentrations of 0.3% (w/v) (B-SLN0.3 and B-SLN0.3W/O). The formulation B-SLN0.3 showed, for all tested times, particle size in the order of 400 nm, always lower ($p<0.05$) concerning that of the non-sonicated sample, which was characterized by a diameter of 520.6±9.73 nm. For B-SLN0.3W/O the use of the sonicator probe did not lead to significant statistical differences ($p>0.05$) both in terms of mean diameter and PDI.

S2.2 Preparation of GEN-loaded SLN

The GEN is a water-insoluble drug, therefore to evaluate the maximum loading capability of SLN the drug precipitation in the formulations aqueous phase was evaluated. In the samples containing higher concentrations of GEN (0.04 and 0.06% w/v) (G-SLNc and G-SLNd, respectively), the formation of a precipitate was observed. The precipitate was analyzed by HPLC and the diode array detection confirmed the characteristic peak of GEN (Figure S4).

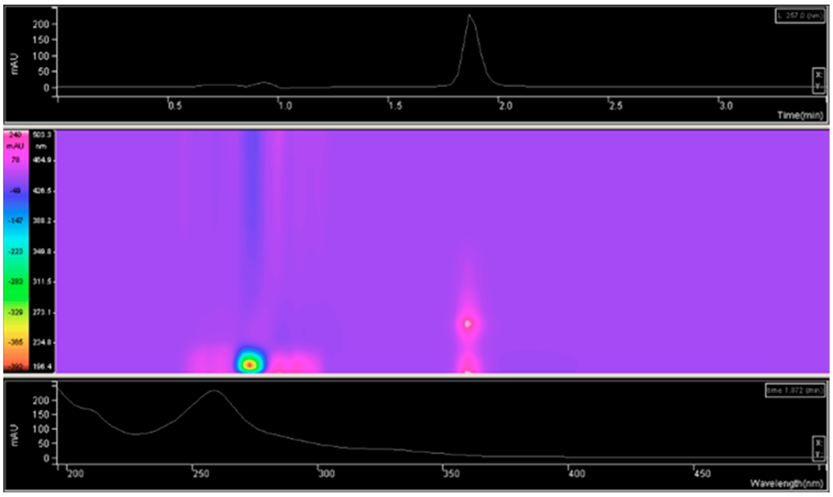

Figure S4. HPLC diode array detection of the precipitate isolated by GEN-loaded SLN with 0.04 and 0.06% (w/v) of the drug. The peak of GEN is evident.

S2.3. Preparation of fluorescein-labeled SLN

Fluorescein-labeled SLN were prepared to assess the intestinal and cellular uptake of nanoparticles. F-SLN showed dimensional properties (mean diameter 253.85 ±8.55 nm; PDI 0.223 ± 0.089) similar to unloaded SLN; furthermore, they were stable in terms of dimensional properties: the particle size and PDI after 10 days were 243.05 ± 1.62 and 0.238 ± 0.057 respectively.

Fluorescein incorporation into SLN is an essential condition to perform the SLN uptake study therefore, the dye incorporation was evaluated by observing its precipitation in the aqueous media of SLN dispersion (Figure S5).

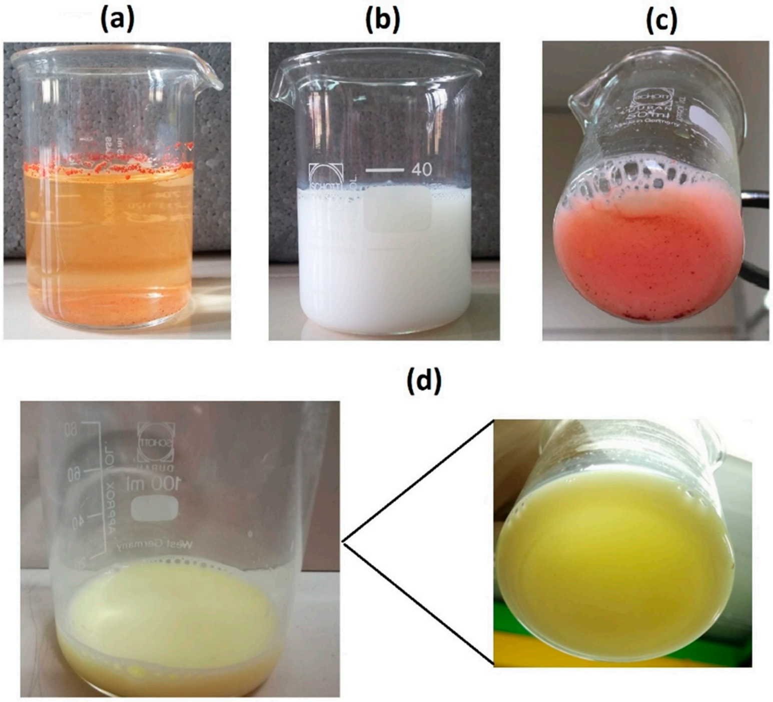

Figure S5. Evaluation of fluorescein incorporation into SLN: water dispersion of free fluorescein (a), blank SLN (b), addition of fluorescein to performed SLN, or addition of free fluorescein to the molten lipid (c), addition of fluorescein solution to the molten lipid (d).

Fluorescein is a slightly water-insoluble day, thus, when it was put in contact with water formed an orange/red precipitate (Figure S5 a). When the fluorescein was added to preformed SLN or as solid to the molten lipid during SLN preparation, the dye incorporation was poor, and the red precipitate of unloaded fluorescein can be easily observed (Figure S5 c). In contrast, the sample F-SLN, obtained by adding an acetone solution of dye during SLN preparation, was yellow, well dispersed, and free from any orange/red precipitate (Figure S5 d) indicating good incorporation of fluorescein into SLN. Besides, no precipitation was observed during the time, consequently, the fluorescein was not released from the SLN. Fluorescence analysis revealed that the fluorescence intensity value of F-SLN did not change during the time (Table S4), showing good stability of fluorescein-labeled SLN.

Table S4. fluorescence stability of F-SLN during the time (2, 1, 5, and 10 days)**.**

Time (days)	Emission wavelength (nm)	Fluorescence intensity (a.u.)
0	514	76939.5
1	514	73176.5
5	514	75649.8
10	514	88136.1

References

1. Müller, R. H.; Mäder, K.; Gohla, S. Solid Lipid Nanoparticles (SLN) for Controlled Drug Delivery - a Review of the State of the Art. *Eur J Pharm Biopharm* **2000**, *50*, 161–177, doi:10.1016/s0939-6411(00)00087-4.
2. Mehnert, W.; Mäder, K. Solid Lipid Nanoparticles: Production, Characterization and Applications. *Adv Drug Deliv Rev* **2001**, *47*, 165–196, doi:10.1016/s0169-409x(01)00105-3.

3. Darwis, Y.; Ali Khan, A.; Mudassir, J.; Mohtar, N. Advanced Drug Delivery to the Lymphatic System: Lipid-Based Nanoformulations. *Int J Nanomedicine* **2013**, 2733-2747, doi:10.2147/IJN.S41521.
4. Müller, R.H.; Rühl, D.; Runge, S.; Schulze-Forster, K.; Mehnert, W. Cytotoxicity of Solid Lipid Nanoparticles as a Function of the Lipid Matrix and the Surfactant. *Pharm Res* **1997**, *14*, 458–462, doi:10.1023/a:1012043315093.
5. Arechabala, B.; Coiffard, C.; Rivalland, P.; Coiffard, L.J.; de Roeck-Holtzhauer, Y. Comparison of Cytotoxicity of Various Surfactants Tested on Normal Human Fibroblast Cultures Using the Neutral Red Test, MTT Assay and LDH Release. *J Appl Toxicol* **1999**, *19*, 163–165, doi:10.1002/(sici)1099-1263(199905/06)19:3<163::aid-jat561>3.0.co;2-h.
6. Vitorino, C.; Carvalho, F.A.; Almeida, A.J.; Sousa, J.J.; Pais, A.A.C.C. The Size of Solid Lipid Nanoparticles: An Interpretation from Experimental Design. *Colloids Surf B Biointerfaces* **2011**, *84*, 117–130, doi:10.1016/j.colsurfb.2010.12.024.
7. Makwana, V.; Jain, R.; Patel, K.; Nivsarkar, M.; Joshi, A. Solid Lipid Nanoparticles (SLN) of Efavirenz as Lymph Targeting Drug Delivery System: Elucidation of Mechanism of Uptake Using Chylomicron Flow Blocking Approach. *Int J Pharm* **2015**, *495*, 439–446, doi:10.1016/j.ijpharm.2015.09.014.
